# Prevalence and Factors Associated with Problematic Internet Use in a Population of Spanish University Students

**DOI:** 10.3390/ijerph18147620

**Published:** 2021-07-17

**Authors:** Enrique Ramón-Arbués, José Manuel Granada-López, Blanca Martínez-Abadía, Emmanuel Echániz-Serrano, Isabel Antón-Solanas, Michael Nash

**Affiliations:** 1 Faculty of Health Sciences, Campus Universitario Villanueva de Gállego, Universidad San Jorge, Villanueva de Gállego, 50830 Zaragoza, Spain; eramon@usj.es; 2 Research Group Transfercult (H27_20D), University of Zaragoza, 50009 Zaragoza, Spain; eechaniz@unizar.es; 3 Department of Physiatrics and Nursing, Faculty of Health Sciences, University of Zaragoza, C/Domingo Miral S/N, 50009 Zaragoza, Spain; 4 Research Group Safety and Care (GIISA021), Institute of Research of Aragón, 50009 Zaragoza, Spain; 5 Occupational Health and Prevention Service, Zaragoza City Council, P° de La Mina 9, 50001 Zaragoza, Spain; bmartinez@zaragoza.es; 6 Research Group Nursing Research in Primary Care in Aragón (GENIAPA) (GIIS094), Institute of Research of Aragón, 50009 Zaragoza, Spain; 7 School of Nursing and Midwifery, Trinity College, University of Dublin, Dublin 2, Ireland; NASHMI@tcd.ie

**Keywords:** problematic Internet use, university students, stress, anxiety

## Abstract

(1) Background: To examine the prevalence, and associated factors of, problematic Internet use in a sample of Spanish university students. (2) Methods: Cross-sectional descriptive study of a convenience sample of 698 university students. Self-esteem, alcohol consumption, perceived social support, depression, anxiety, stress and problematic Internet use were evaluated using the Rosenberg, CAGE, DUKE-UNC-11, DASS-21 and Young’s Internet Addiction Test, respectively. (3) Results: Problematic internet use was reported by 21% of respondents. Risk of problematic Internet use was independently associated with the preferred use of the smartphone, time of exposure to the Internet, less perceived social support, problematic alcohol consumption and symptoms of stress and anxiety. We found significant association between problematic internet use and time of exposure to the Internet, residential status, alcohol consumption, self-esteem, perceived social support and psychological distress, after bivariate analysis. (4) Conclusions: A considerable prevalence of problematic Internet use was found; in our sample problematic Internet use was associated with stress, alcohol consumption, anxiety and perceived social support. Strategies aimed at the early identification of problematic Internet use may lead to an improvement in the psychosocial health of the university student population.

## 1. Introduction

The number of Internet users has grown exponentially in the world [1]. It is estimated that the percentage of users in developed countries, such as North America, Europe or East Asia, ranges between 70 and 90% of the population [2] and the number of active users in the world far exceeds 4 billion people [3]. The Internet provides modern society with many possibilities in education, science, information and communication, entertainment or removal of barriers, among other things. However, sometimes its use is accompanied by negative effects and misuse. Research on disorders related to the use of the Internet began to appear in the biomedical literature in the 1990s but, still today, researchers use different terms and definitions when conceptualizing the problems associated with Internet use. In this way, terms such as “Internet addiction”, “pathological use of the Internet”, “problematic use of the Internet”, “compulsive use of the Internet”, “cyberdiction” or “netting”, among others, coexist in the available literature [4,5], making it difficult to compare the results of different studies. For this study we will use the term “Problematic Internet Use”, abbreviated to PIU as the descriptor.

In addition to the difficulties mentioned above, recent empirical research on Internet use has not been able to solve the issue of the classification and diagnosis of PIU, neither has it produced a gold-standard instrument to assess it. Instead, current PIU assessment tools are based on the following dimensions of addiction: compulsive use, negative outcome, salience, withdrawal symptoms, mood regulation, escapism and social discomfort [6], all frequent components of behavioural addition [7].

From a neurobiological point of view, PIU is characterised by impulsivity [8] and alterations in the reward [9] and dopaminergic [10] systems, both usually related to addictions. Additionally, Leet et al. [11] detected a higher probability of genetic variation in serotonin transporters in adolescents engaging in PIU.

The factors and comorbidities most frequently associated with disorders related to PIU have been the presence of certain psycho-social variables such as depression, stress and anxiety [12,13,14], male gender [15,16], substance use [17] or lack of social support [18]. In addition, age has shown an inverse association with disorders related to Internet use [2,19,20].

Online gaming and social media are the most common causes of PIU [21,22]. Other factors including availability of devices, amount of time in the Internet [23,24] and family disfunction and conflict [25,26] seem to also contribute to PIU. Several studies [8,22,27,28] have identified specific personality traits and behaviours associated with PIU, including high exploratory excitability, impulsive sensation-seeking, hostility, low self-esteem and isolation. Similarly, some authors [29,30] have suggested that specific pre-existing mental health problems, such as hostility and attention-deficit/hyperactivity disorder (ADHD), can increase vulnerability to PIU. Finally, previous studies [31,32] suggest that abuse of alcohol and other substances often precedes PIU.

The prevalence of Internet-related disorders varies greatly depending on the investigation method used and the population under study. In the United States, the prevalence Internet-related disorders ranges from 0.7% to 26.3% in adolescents and university students [33]. In Europe, a cross-sectional study conducted on 11.356 adolescents in 11 countries places the prevalence of non-adaptive Internet use at 17.6% [34]. In the general European population, the prevalence ranges from 1% to 18.3% [35,36]. In Spain, research on the subject is still scarce and fundamentally directed towards primary and secondary school populations [37,38]. In addition, this research is not entirely applicable to the population of college students due to differences in age, educational and socio-economic level and academic requirements of Internet use. However, although research on Internet use in the Spanish college student population is very limited, it points at a concerning increase in the prevalence of addiction to smartphones, Internet and social media in the past 15 years [39]. Specifically, 1 in 10 college students seems to meet the criteria for PIU, especially in association with male gender, amount of time spent in the Internet and high level of interaction with other people online [20,40]. Given the lack of evidence regarding PIU in the population of college students, we aimed to examine and evaluate the prevalence, characteristics, patterns of Internet use and risk factors in a young adult population of Spanish university students at the San Jorge University Campus in Zaragoza (Spain), as well as its associated factors.

## 2. Materials and Methods

### 2.1. Design and Study Population

A cross-sectional descriptive design was employed for this study. The population was a convenience sample of university students on different under-graduate degree programmes at the San Jorge University campus in Zaragoza, Spain. Participants were recruited in the classroom during the first four-month period of the 2019–2020 academic year. All participants were informed of the objectives of the research at the beginning of one of their classes. Questionnaires were administered at the end of the class. During the data collection period, which lasted for 4 weeks, a total of 811 students were invited to participate in the study. A total of 698 (417 women and 281 men) students gave their consent to enter this investigation and completed the requested questionnaire as requested.

### 2.2. Data Collection

Data was collected on demographics such as age, gender, bachelor’s degree, residential status, relationship status, smoking status, daily use of Internet and physical parameters including body mass index (BMI). With regard to BMI, we classified our participants according to the following criteria: low weight (<18.5 kg/m^2^), normal weight (18.5–24.9 kg/m^2^) y overweight/obese (≥25 kg/m^2^) [41]. In addition, we collected information about PIU risk factors previously described in the literature, namely alcohol use [42,43], self-esteem [44,45], social support [46,47], depression [47,48], anxiety [49] and stress [15,48].

The evaluation of Internet addiction symptoms was tested through Young’s Internet Addiction Test (IAT), validated in Spanish by Puerta-Cortés et al. [50] in 2012. Cronbach’s alpha for this tool in its Spanish version was high (α = 0.89), and it was similar to previous validation studies of the instrument [51]. This tool consists of 20 items rated on a Likert scale of 1 to 5 points. The maximum score to be obtained is 100. Scores <50 points have been associated with controlled Internet users, from 50 to 79 points to problematic Internet users (PIU) and ≥80 points to significant vital problems arising from the use of the Internet [4]. In this study the result of the questionnaire has been dichotomized into—No PIU (IAT < 50) and PIU (IAT ≥ 50) [15,52].

Alcohol consumption was assessed through the CAGE questionnaire, validated in the Spanish population by Rodríguez Martos et al. [53]. This questionnaire consists of 4 dichotomous response items (Yes/No). Each affirmative item adds a point, considering that there are problems with alcohol when there is an affirmative answer to 2 or more questions. Its sensitivity ranges in 65–100% and the specificity in 88–100% [54].

The Rosenberg scale was used to assess the self-esteem of the participants. This questionnaire consists of 10 Likert items with 1 to 4 points that lead to a minimum result of 10 and a maximum of 40 points. The classification of the participants’ self-esteem meets the following criteria: <25 points (low self-esteem); 26–29 points (average self-esteem) and ≥30 points (high self-esteem). In the Spanish population, this scale has obtained an internal consistency of 0.87 and a test–retest reliability of 0.74 in one year [55].

The symptoms related to anxiety, stress and low mood/depression of the participants were assessed using the DASS-21 questionnaire, short version of the DASS-42. DASS-21 is made up of the DASS-A (anxiety), DASS-S (stress) and DASS-D (depression) subscales. DASS-21 is an instrument composed of 21 items, 7 for each subscale, with a Likert evaluation of 0 to 3 points (0 means “does not apply to me at all” and 3 “applies to me a lot or most of the time”). The sum of the scores obtained in each subscale is multiplied by 2 in order to make the results of DASS-21 and DASS-42 comparable. Based on the obtained scores, the participants are classified in each of the 3 subscales as follows:

- Anxiety: Normal (0–7 points), mild (8–9), moderate (10–14), severe (15–19) and extremely severe (>19).
- Depression: Normal (0–9 points), mild (10–13), moderate (14–20), severe (21–27) and extremely severe (>27).
- Stress: Normal (0–14 points), mild (15–18), moderate (19–25), severe (26–33) and extremely severe (>33).

The DASS-21 questionnaire has been previously validated in the Spanish university population with internal consistency values for the three subscales that ranged between 0.73 and 0.81 [56].

The perceived social support was assessed using the DUKE-UNC-11 questionnaire from Broadhead et al. [57]. This is a questionnaire consisting of 11 items and a Likert response scale of 1 to 5 points. The scoring range ranges from 11 to 55 points. The lower the score, the less support. In the Spanish validation, a cut-off point in the 15^th^ percentile was chosen, corresponding to a score <32. Thus, a score ≥32 indicates normal support, while less than 32 indicates low perceived social support [58].

### 2.3. Data Analysis

The characteristics of the sample were summarized using mean and standard deviation for continuous variables and frequency and percentage for nominal data.

The Kolmogorov–Smirnov test was used to test the normality of the distributions of each variable. The bivariate analysis was performed using the Chi-square test and U Mann–Whitney tests, as appropriate, as well as bivariate correlation analysis, tested through the Spearman correlation test, between continuous variables. In addition, a binary logistic regression analysis was performed in order to determine the factors independently associated with PIU (IAT score ≥ 50). Multivariate model was carried out by the backward stepwise method with a probability value for the entry of *p* = 0.05 and removal of *p* = 0.10. In addition, the collinearity between independent variables was tested in order to exclude those highly correlated (condition index ≥ 30). The statistical analysis of the data was performed with the SPSS statistical package for Windows (version 21, IBM, Chicago, IL, USA) accepting a significance level of *p* < 0.05.

## 3. Results

The final sample size was 698 participants, with a predominance of women (59.7%) and a mean age of 21.96 ± 5.43. Sample characteristics are summarized in Table 1.

The average score in the IAT was 41.68 ± 9.09. Average Internet exposure was 4.95 ± 2.72 h and 148 (21.2%) of the students were classified as having PIU. Smartphones were the preferred connection device. Smoking prevalence was 23.4% and 33.0% reported a problematic alcohol consumption assessed by the CAGE. Low social support was reported by 4.2% of respondents and 9.5% showed low self-esteem. Finally, results obtained through the DASS-21 questionnaire showed that 31.9%, 18.6% and 22.6% of the participants suffered some degree of stress, depression or anxiety, respectively (see Table 1).

In the bivariate analysis (see Table 2), the probability of having PIU was significantly related to the time of exposure to the Internet, the residential status, alcohol consumption, self-esteem, perceived social support and psychological distress (*p* < 0.01 for all the comparisons).

In addition, the study of bivariate correlations showed significant associations (*p* < 0.01), from weak to moderate, between all the study variables with the exception of alcohol consumption, assessed through the CAGE questionnaire, which was not associated with symptoms of stress and anxiety as measured by the DASS-S and DASS-A (see Table 3).

The logistic regression analysis (see Table 4) showed that the risk of having PIU is independently associated with the preferred use of the smartphone (OR = 3.399 (1.512–7.643)), the time of exposure to the Internet (OR = 1.248 (1.131–1.378)), the symptoms of stress (OR = 1.115 (1.072–1.159)) and anxiety (OR = 1.060 (1.006–1.117)), and alcohol consumption (OR = 1.916 (1.382–2.656)). On the contrary, female gender, living alone and perceived social support were inversely associated (OR = 0.881 (0.840–0.924)).

## 4. Discussion

The objective of our research was to determine the prevalence and characteristics of PIU in a Spanish university campus, and its possible association with psychological symptomatology. The prevalence of PIU was 21.2% (19.4% in women and 23.8% in men). Similar prevalence rates have been reported in the university population of different countries. In American university students PIU prevalence was 25.1% [59], in Malaysian students 36.9% [60], in Iranian students 40.7% [61], in Japanese students 38.2% [62], in Chinese students 9.2% [63] and 9.7% in Indian students [16]. Notwithstanding the possible variations of device penetration and access to the Internet in these countries, it should be noted that the variability of these results can be explained by the different cut-off points in the IAT considered by the authors (≥40, 43, 40, 40, 50 and 50, respectively). Our cut-off point was IAT ≥ 50.

In our sample, the smartphone was the preferred device to access the Internet (91.0%). This is significant, as mobile phones are not usually linked to academic work but to leisure activities and social interaction. Thus, we argue that smartphone addiction may be more associated with social support [64] and pleasure derived from social interaction than Internet use alone.

The factors independently associated with the probability of having PIU were male gender, daily time connected to the Internet, perceived social support, depression and anxiety symptoms, alcohol consumption and the preferred use of the smartphone to connect to the network. The male gender has traditionally been linked to disorders related to the use of the Internet [14,15,16,65]. It has been argued that young men are more interested in information and communication technologies (for example, playing online games, engaging in cybersex and gambling) and thus spend more time using the Internet, than young women [66,67]. However, other authors have not found gender differences [20] or have even observed a higher prevalence of PIU in their cohort of women [62].

As in our sample, several studies have reported associations between PIU and lack of perceived support [18,19], alcohol consumption [31,68], anxiety states [15,34,62] and stress [15,48], preferred use of the smartphone [65,69] or time spent browsing the net [14,16]. Peer to peer relationships are essential to satisfy the social needs of adolescents and young adults. In addition, they play a key role in the emotional and social development of these groups. When adolescents and young adults experience a low degree of social support and are unable to establish close physical connections, they may resort to the Internet to satisfy their social needs, or to develop alternative social relationships [46].

The association between PIU and other associated health problems, including impulse control disorders and behavioural addictions, have previously been reported in the literature [10]. Some of these behaviours may provide a reward in the short-term. If, despite the possible negative effects, this leads to a decrease in behavioural control, then that behaviour becomes a source of addiction instead of a psychoactive substance [70,71]. In addition, behavioural or non-substance addictions (such as Internet addiction) are similar to substance addictions from both a phenomenological and neurobiological point of view. Behavioural addictions usually begin during adolescence and early adulthood, to become chronic (with remissions and exacerbations) later on in life [72]. Adolescence and early adulthood are vulnerable periods for addictive behaviours, as social demands (frequently through social media) are high and risk behaviours are expected and even promoted [73].

We observed a significant association between depressive symptoms and the IAT total score after bivariate analysis. However, we could not confirm this relationship after multivariate logistic regression analysis, as opposed to previous studies [14,15,16,34]. This may be due to two factors. First, our logistic regression analysis was done on the premise that the score from the DASS-D subscale was a quantitative variable. It is possible that a different codification, based on a classification of the degree of severity of the symptoms for example, produced different results. Second, there may exist confounding variables affecting the relationship between depressive symptoms and PIU. Specifically, both perceived social support [47] and sleep quality [74,75] were not measured in our study. It is possible that individuals that present PIU spend a greater amount of time online, which may impact on their sleep–wake rhythm and, ultimately, contribute to the appearance of depressive symptoms [76,77].

Managing “screen time” has excised parents, healthcare practitioners and policy makers in the area of child health. However, as adults, such paternalistic approaches are unlikely to work with university students. Instead, we need to look at strategies that 1) aim to prevent PIU arising, e.g., general public health type messages, 2) support students who have a PIU, e.g., mindfulness and resilience skills so that they can develop diversion strategies, and 3) help with any underlying mental health conditions that may be present with PIU. To this end, new investigations of a longitudinal nature are necessary in order to determine any causal relationship between PIU and the co-occurrence of associated mental health symptoms. This would include the treatment of PIU.

This study has several limitations. The cross-sectional design does not allow us to draw conclusions about cause-and-effect relationships within the data. The convenience sample may not allow for generalisation to the wider student population. The method of recruitment may introduce a selection bias where those with more interest in the area may participate in the study while those that may have a PIU may not. However, with anonymous participation it is possible that these methods more faithfully reflect the participants’ own perspective and, therefore, can better adjust to the reporting of subjective disorders such as low self-esteem, low perceived social support or certain psychological symptomatology [15]. In addition, this study does not investigate the Internet content accessed by the participants (e.g., gaming activity, social networks, online academic learning). This could explain in some way the classification of PIU, e.g., if student learning is based online then frequent accessing of the Internet may be an artefact of the educational course rather than PIU. Thus, we recommend that future investigations include an analysis of college students’ preferred online content and access. Finally, it should be noted that the IAT questionnaire was developed more than 20 years ago, when access and use of the Internet was much lower and less restricted than today, and that it does not evaluate the presence of symptoms based on a specific period of time. However, despite these limitations, the IAT continues to be the most frequently used tool in the assessment and screening of PIU. Further, the psychometric properties of this instrument are very satisfactory [22], even across different cultures [78].

In spite of these limitations, our results can potentially serve as a starting point for new investigations in this field and inform future health promoting interventions. To our knowledge, this is the first study to address the prevalence of PIU, as well as its relationship to a range of sociodemographic behavioural and psychological variables, in a large sample of Spanish college students. Our findings highlight the importance of establishing an early diagnosis and treatment in this population as PIU often coexists with other psychological disorders and risk behaviours.

## 5. Conclusions

Our findings denote a considerable prevalence of PIU in our sample population. PIU was associated with perceived stress, anxiety, alcohol consumption and perceived social support. From the point of view of public health, strategies aimed at the early identification of PIU can lead to greater psychosocial well-being of the young adult population. For students entering university life we feel that as part of their healthcare or student support services, assessment of PIU should be encouraged by student healthcare services.

## Figures and Tables

**Table 1:** Participant characteristics (*n* = 698), number, % or mean ± SD.

Variable	Category	*n*(%) or *n* ± SD
Age		21.96 ± 5.43
Gender	Female	417 (59.7%)
	Male	281 (40.3%)
BMI (Kg/m^2^)		22.45 ± 3.63
BMI Categories	Under weight	104 (14.9%)
	Normal weight	471 (67.5%)
	Over-weight/obese	123 (17.6%)
Degree	Health related	258 (37.0%)
	Non health related	440 (63.0%)
Residential status	Living alone	48 (6.9%)
	Living with a partner	193 (27.7%)
	Living with family	457 (65.5%)
Relationship status	Currently in a relationship	305 (43.7%)
	Not currently in a relationship	393 (56.3%)
Smoking status	No	535 (76.6%)
	Yes	163 (23.4%)
Hours/day of Internet use		4.95 ± 2.72
Preferred device	Smartphone	635 (91.0%)
	PC/Tablet	63 (9.0%)
IAT Score		41.68 ± 9.09
	No PIU	550 (78.8%)
	PIU	148 (21.2%)
CAGE Score		0.48 ± 0.78
CAGE Categories	Problematic alcohol consumption	230 (33.0%)
	No problematic alcohol consumption	468 (67.0%)
DASS-S score		12.05 ± 7.88
DASS-S categories	No stress	475 (68.1%)
	Mild stress	77 (11.0%)
	Moderate stress	110 (15.8%)
	Severe stress	27(3.9%)
	Extremely severe stress	9 (1.3%)
DASS-D score		5.45 ± 7.30
DASS-D categories	No depression	568 (81.4%)
	Mild depression	56 (8.0%)
	Moderate depression	26 (3.7%)
	Severe depression	26 (3.7%)
	Extremely severe depression	22 (3.2%)
DASS-A score		4.65 ± 5.51
DASS-A categories	No anxiety	540 (77.4%)
	Mild anxiety	54 (7.7%)
	Moderate anxiety	64 (9.2%)
	Severe anxiety	5 (0.7%)
	Extremely severe anxiety	35 (5.0%)
Rosenberg score		32.12 ± 5.44
Rosenberg categories	High self-esteem	523 (74.9%)
	Half	109 (15.6%)
	Low	66 (9.5%)
DUKE-UNC-11 score		47.03 ± 6.74
DUKE-UNC-11 categories	Low perceived social support	29 (4.2%)
	Normal perceived social support	669 (95.8%)

**Table 2:** Participant characteristics according to Internet use (number, % or mean ± SD.).

Variables	Categories	No PIU (*n* = 550)	PIU (*n* = 148)	Z/*X*^2^	*p*
IAT Score		38.34 ± 6.91	54.08 ± 4.06	−18.70	<0.01 ^a^
Age		22.02 ± 5.17	21.74 ± 6.31	−3.99	<0.01 ^a^
Gender	Female (*n* = 417)	336 (80.6%)	81 (19.4%)	1.40	0.16 ^b^
	Male (*n* = 281)	214 (76.2%)	67 (23.8%)		
BMI (Kg/m^2^)		22.39 ± 3.35	22.66 ± 4.53	−0.72	0.477 ^a^
BMI Category	Underweight (*n* = 104)	74 (71.2%)	30 (28.8%)	5.55	0.062 ^b^
	Normal BMI (*n* = 471)	382 (81.1%)	89 (18.9%)		
	Overweight/Obese (*n* = 123)	94 (76.4%)	29 (23.6%)		
Degree	Health related degree (*n* = 258)	198 (76.7%)	60 (23.3%)	−1.01	0.310 ^b^
	Non health related degree (*n* = 440)	352 (80.2%)	88 (19.8%)		
Habitation status	Living alone (*n* = 48)	44 (91.7%)	4 (8.3%)	22.32	<0.01 ^b^
	Living with fellow students (*n* = 193)	170 (88.1%)	23 (11.9%)		
	Living with family (*n* = 457)	336 (73.5%)	121 (26.5%)		
Relationship status	Currently in a relationship (*n* = 305)	244 (80.0%)	61 (20.0%)	0.47	0.515 ^b^
	Not currently in a relationship (*n* = 393)	306 (77.9%)	87 (22.1%)		
Smoker	No (*n* = 535)	426 (79.6%)	109 (20.4%)	0.97	0.331 ^b^
	Yes (*n* = 163)	124 (76.1%)	39 (23.9%)		
Time spent on Internet (h/day)		4.59 ± 2.59	6.28 ± 2.81	−6.55	<0.01 ^a^
Preferred device	Smartphone (*n* = 635)	507 (79.8%)	128 (20.2%)	2.14	0.032 ^b^
	PC/Tablet (*n* = 63)	43 (68.3%)	20 (31.7%)		
CAGE Score		0.40 ± 0.70	0.79 ± 0.97	−4.79	<0.01 ^a^
CAGE Category	Non-problematic consumption (*n* = 468)	390 (83.3%)	78 (16.7%)	4.18	<0.01 ^b^
	Problematic consumption (*n* = 230)	160 (69.6%)	70 (30.4%)		
DASS-S Score		10.45 ± 7.01	17.98 ± 8.11	−9.89	<0.01 ^a^
DASS-S Category	No stress (*n* = 475)	412 (86.7%)	63 (13.3%)	77.71	<0.01 ^b^
	Mild stress (*n* = 77)	57 (74.0%)	20 (26.0%)		
	Moderate stress (*n* = 110)	68 (61.8%)	42 (38.2%)		
	Severe stress (*n* = 27)	9 (33.3%)	18 (66.7%)		
	Extremely severe stress (*n* = 9)	4 (44.4%)	5 (55.6%)		
DASS-D Score		4.69 ± 7.43	8.27 ± 5.99	−10.06	<0.01 ^a^
DASS-D Category	No depression (*n* = 568)	479 (84.3%)	89 (15.7%)	89.78	<0.01 ^b^
	Mild depression (*n* = 56)	24 (42.9%)	32 (57.1%)		
	Moderate depression (*n* = 26)	8 (30.8%)	18 (69.2%)		
	Severe depression (*n* = 26)	21 (80.8%)	5 (19.2%)		
	Extremely severe depression (*n* = 22)	18 (81.8%)	4 (18.2%)		
DASS-A Score		3.66 ± 4.55	8.31 ± 7.04	−9.18	<0.01 ^a^
DASS-A Category	No anxiety (*n* = 540)	463 (85.7%)	77 (14.3%)	106.41	<0.01 ^b^
	Mild anxiety (*n* = 54)	20 (37.0%)	34 (63.0%)		
	Moderate anxiety (*n* = 64)	49 (76.6%)	15 (23.4%)		
	Severe anxiety (*n* = 5)	0 (0.0%)	5 (100.0%)		
	Extremely severe anxiety (*n* = 35)	18 (51.4%)	17 (48.6%)		
Rosenberg Score		32.84 ± 5.39	29.45 ± 4.75	−7.87	<0.01 ^a^
Rosenberg Category	High self-esteem (*n* = 523)	445 (85.1%)	78 (14.9%)	52.20	<0.01 ^b^
	Moderate self-esteem (*n* = 109)	61 (56.0%)	48 (44.0%)		
	Low self-esteem (*n* = 66)	44 (66.7%)	22 (33.3%)		
DUKE-UNC-11 Score		47.67 ± 6.20	44.65 ± 8.04	−4.40	<0.01 ^a^
DUKE-UNC-11 Category	Low Perceived Social Support (*n* = 29)	14 (48.3%)	15 (51.7%)	−4.10	<0.01 ^b^
	Normal Perceived Social Support (*n* = 669)	536 (80.1%)	133 (19.9%)		

^a^ U Mann–Whitney test; ^b^ chi square test.

**Table 3:** Spearman correlation coefficients between the results of the questionnaires (*n* = 698).

	IAT	DASS-S	DASS-D	DASS-A	Rosenberg	DUKE-UNC-11	CAGE
IAT	1						
DASS-S	0.392 *	1					
DASS-D	0.404 *	0.488 *	1				
DASS-A	0.244 *	0.500 *	0.559 *	1			
Rosenberg	−0.332 *	−0.355 *	−0.624 *	−0.436 *	1		
DUKE-UNC	−0.311 *	−0.108 *	−0.288 *	−0.163 *	0.440 *	1	
CAGE	0.158 *	0.069	0.215 *	0.030	−0.196 *	−0.278 *	1

* *p* < 0.01 (bilateral).

**Table 4:** The logistic regression analysis of the risk of having a PIU.

							I.C. 95% OR		
Independent Variables	B	E.T.	Wald	gl	Sig.	OR	Lower	Upper
Female	−1.256	0.334	14.093	1	0.000	0.285	0.148	0.549
Time spent on Internet (h/day)	0.222	0.050	19.443	1	0.000	1.248	1.131	1.378
Living alone	−1.348	0.291	21.457	1	0.000	0.260	0.147	0.459
Preferred device Smartphone	1.224	0.413	8.761	1	0.003	3.399	1.512	7.643
DASS-S	0.108	0.020	30.246	1	0.000	1.115	1.072	1.159
DASS-D	−0.031	0.022	1.957	1	0.162	0.969	0.928	1.013
DASS-A	0.058	0.027	4.689	1	0.030	1.060	1.006	1.117
Rosenberg	0.035	0.030	1.372	1	0.242	1.036	0.977	1.099
DUKE-UNC-11	−0.127	0.024	27.619	1	0.000	0.881	0.840	0.924
CAGE	0.650	0.167	15.202	1	0.000	1.916	1.382	2.656
Constant	0.865	1.240	0.486	1	0.486	2.375		

## Data Availability

The data presented in this study are available on request from the corresponding author. The data are not publicly available as they contain information that could compromise the privacy of research participants.
